# A new species of *Ptilomymar* (Hymenoptera, Mymaridae) and a key to the described species

**DOI:** 10.3897/zookeys.439.8304

**Published:** 2014-09-10

**Authors:** Xiang-Xiang Jin, Cheng-De Li

**Affiliations:** 1 School of Forestry, Northeast Forestry University, Harbin, 150040, China

**Keywords:** Chalcidoidea, Mymaridae, *Ptilomymar dianensis*, taxonomy, new species, China

## Abstract

*Ptilomymar dianensis* **sp. n.** (Hymenoptera, Mymaridae) from southwest China is described and illustrated. A key to the six described species is given. The type specimens are deposited in the insect collections of Northeast Forestry University, China.

## Introduction

*Ptilomymar* was established by Annecke and Doutt (1961). Currently, this genus contains five described species, *Ptilomymar rete* Annecke & Doutt from Mexico, *Ptilomymar orientalis* Taguchi from the Philippines (Taguchi, 1972), *Ptilomymar besucheti* Viggiani from Sri Lanka (Viggiani, 1974), *Ptilomymar magnificum* Yoshimoto from Canada (Yoshimoto 1990), and *Ptilomymar dictyon* Hayat & Anis from India (Hayat and Anis 1999). Here we describe a new species of *Ptilomymar* from southwest China. A tentative key to species is provided based on their original descriptions. No types other than that of the new species were examined.

## Materials and methods

Specimens were collected from Yunnan Province (southwest China) using yellow pan traps. Specimens were dissected and mounted dorsally or laterally in Canada balsam on slides following the method described by Noyes (1982) and modified for the Mymaridae by Huber (1988). Photographs were taken with a digital CCD camera attached to an Olympus BX51 compound microscope, and most measurements were made from slide-mounted specimens using an eye-piece reticle. Total body length excluding ovipositor was measured with an eye-piece reticle from alcohol-preserved specimens before being dissected. All measurements are given in micrometers (μm). Specimens studied are deposited in the following institution:

NEFU Northeast Forestry University, Harbin, China.

Morphological terminology and abbreviations are those of Gibson (1997) and Huber (2012), as follows (with some additions):

OD Mid ocellar diameter

OOL Ocular-ocellar length

LOL Least ocellar length

POL Postocellar length

Fln Flagellar segment

Gtn Gastral tergum

## Results

### Key to species of *Ptilomymar* of the world (based on features from the original descriptions and illustrations).

(Note: females are not known for *orientalis*; males are not known for *dictyon* and *rete*)

**Table:** 

1	♀: flagellum clavate, funicle 8-segmented and clava 1-segmented	2
−	♂: flagellum filiform, 11-segmented	6
2	Scape distinctly enlarged ventrally in apical half (Fig. 1)	3
−	Scape not distinctly enlarged ventrally in apical half	4
3	Pedicel about 1.6× as long as fl_1_; fl_1_ distinctly longer than wide (Fig. 1); fore wing about 3.6× as long as wide, with a triangular dark brown marking behind marginal vein (Fig. 4); metanotum about 0.25× as long as scutellum	*Ptilomymar dianensis* sp. n.
−	Pedicel about 5.0× as long as fl_1_; fl_1_ as long as or at most slightly longer than wide; fore wing about 5.4× as long as wide, without a broad dark band behind marginal vein; metanotum slightly less than 0.5× as long as scutellum	*Ptilomymar magnificum*
4	Propodeum with strong reticulations lateral to the translucent carinae; petiole not much longer than wide; gt_1_ with small translucent carinae	*Ptilomymar rete*
−	Propodeum almost smooth lateral to the translucent carinae; petiole at least 2× as long as wide; gt_1_ with large translucent carinae	5
5	Fl_7_ and fl_8_ each distinctly shorter than fl_3–6_ individually; gt_1_ with a pair of scale-like setae on each side; ovipositor not exserted	*Ptilomymar dictyon*
−	Fl_3–8_ almost subequal in length; gt_1_ without scale-like setae; ovipositor distinctly exserted	*Ptilomymar besucheti*
6	Propodeum with unbranched spiracular setae	*Ptilomymar orientalis*
−	Propodeum with branched spiracular setae	7
7	Scape distinctly enlarged ventrally in apical half (Fig. 10)	8
−	Scape not distinctly enlarged ventrally in apical half	*Ptilomymar besucheti*
8	Pedicel about 1.3× as long as fl_1_; fl_1_ distinctly longer than wide; fore wing with a triangular dark brown marking behind marginal vein (Fig. 11); metanotum 0.25× as long as scutellum	*Ptilomymar dianensis* sp. n.
−	Pedicel about 3.0× as long as fl_1_; fl_1_ as long as or at most slightly longer than wide; fore wing without a broad dark band behind marginal vein; metanotum slightly less than 0.5× as long as scutellum	*Ptilomymar magnificum*

### Ptilomymar dianensis

Taxon classificationAnimaliaHymenopteraMymaridae

Jin & Li sp. n.

http://zoobank.org/457CE7F5-F306-410B-BE28-E46C5D092CCB

Figs 1 – 12

#### Holotype

♀ (NEFU), China, Yunnan Province, Mengla County, Menglun Town, Mannanxing, 11–13.I. 2013, Hui-Lin Han, Ye Chen.

#### Paratypes.

**Two males. CHINA.** Yunnan. Same data as holotype (1♂, NEFU); Jinghong City, Yexianggu, 17–18.I. 2013, Hui-Lin Han, Ye Chen (1♂, NEFU).

#### Diagnosis.

Scape distinctly enlarged ventrally in apical half; pedicel about 1.6× as long as fl_1_; fl_1_ distinctly longer than wide; fore wing 3.62× as long as wide, with a triangular dark brown marking behind marginal vein, and a narrow brown strip just beyond venation; gt_1_ with large translucent carinae; ovipositor distinctly exserted.

*Ptilomymar dianensis* is distinguished from most other species except *Ptilomymar magnificum* by the shape of the scape that is distinctly enlarged ventrally in apical half (the scape not distinctly enlarged ventrally in apical half in the remaining species), *Ptilomymar dianensis* differs from *Ptilomymar magnificum* by its longer fl_1_ (shorter in *Ptilomymar magnificum*), wider fore wing (narrower in *Ptilomymar magnificum*), and shorter metanotum, 0.25× as long as scutellum (longer metanotum, slightly less than 0.5× as long as scutellum in *Ptilomymar magnificum*). *Ptilomymar dianensis* differs from *Ptilomymar rete* by its larger translucent carinae (smaller in *Ptilomymar rete*) and distinctly exserted ovipositor (not distinctly exserted in *Ptilomymar rete*). *Ptilomymar dianensis* differs from *Ptilomymar orientalis* by its branched spiracular setae on propodeum (unbranched spiracular setae in *Ptilomymar orientalis*), wider fore wing (narrower in *Ptilomymar orientalis*), and larger facets (smaller in *Ptilomymar orientalis*). *Ptilomymar dianensis* differs from *Ptilomymar besucheti* and *Ptilomymar dictyon* by its longer fl_1_ (shorter in the latter two), wider fore wing (narrower in the latter two), distinctly exserted ovipositor (not exserted in *Ptilomymar dictyon*), fl_3–8_ almost subequal in length (fl_7_ and fl_8_ each distinctly shorter than fl_3–6_ individually in *Ptilomymar dictyon*).

Description. Female. Head dark brown with ocelli black. Antenna brown with fl_1_ slightly lighter, scape and pedicel yellowish-brown. Mesosoma dark brown with pronotum and petiole brown. Fore wing hyaline, with a triangular dark brown marking behind marginal vein, and a narrow brown strip just beyond venation. Venation brown with stigmal vein dark brown. Legs yellowish-brown with last tarsal segments brown. Metasoma dark brown with ovipositor brown.

**Figures 1–3.**
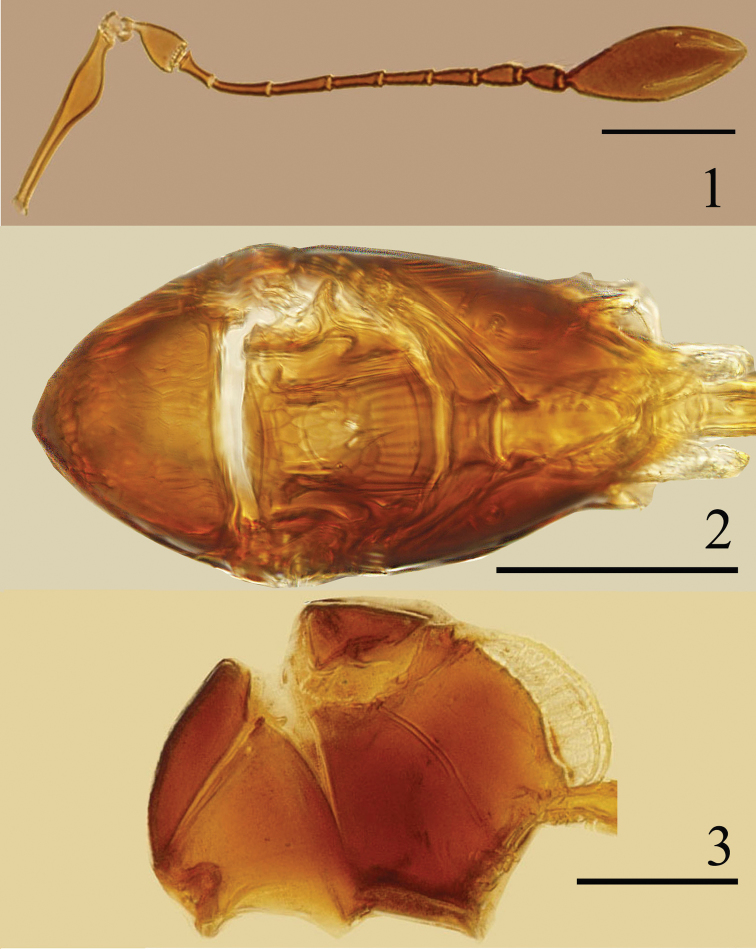
*Ptilomymar dianensis* sp. n., holotype female: **1** antenna **2** mesosoma, dorsal **3** mesosoma, lateral. Scale bars=100 μm.

Head. Eye about 1.5× as long as wide; facets large, each nearly the size of an ocellus. Vertex 0.82× as long as wide, with strong reticulate sculpture; POL about 6.5× as long as OOL. Antenna (Fig. 1). Scape 5.45× as long as wide, longitudinally striate, distinctly enlarged ventrally in apical half; pedicel almost smooth, 1.31× as long as wide, and 1.55× as long as fl_1_; fl_1_ distinctly longer than wide; fl_2_ slightly longer than pedicel, 1.64× as long as fl_1_; clava 2.48× as long as wide.

Mesosoma (Fig. 2) 1.95× as long as wide. Mesoscutum 0.58× as long as wide, with strong reticulation. Scutellum with strong reticulation on anterior scutellum and longitudinal striate on posterior scutellum; with a pair of campaniform sensilla nearer posterior margin than anterior margin. Metanotum 0.25× as long as scutellum. Mid panel of metanotum subrectangle, with longitudinal striate. Propodeum slightly shorter than mesoscutum, without reticulate sculpture, with 2 large subparallel translucent carinae (Figs 2, 3, 6, 7) and 2 branched setae, each on lateral to spiracle.

**Figures 4–7.**
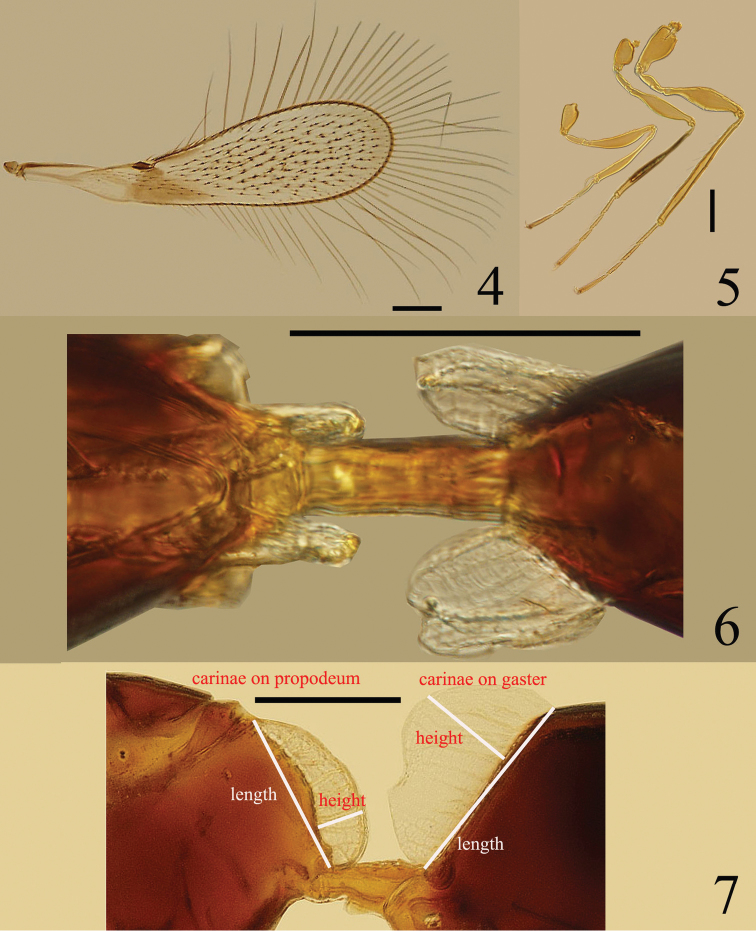
*Ptilomymar dianensis* sp. n., holotype female: **4** fore wing **5** legs **6** carinae on mesosoma and metasoma, dorsal **7** carinae on mesosoma and metasoma, lateral. Scale bars=100 μm.

Fore wing (Fig. 4) 3.62× as long as wide, longest marginal setae 1.38× as long as greatest wing width. Stigmal vein with 4 campaniform sensilla apically.

Legs (Fig. 5) with femora, especially metafemur, swollen medially. Mesocoxa without teeth-like structures on the posterior surface.

**Figures 8–12.**
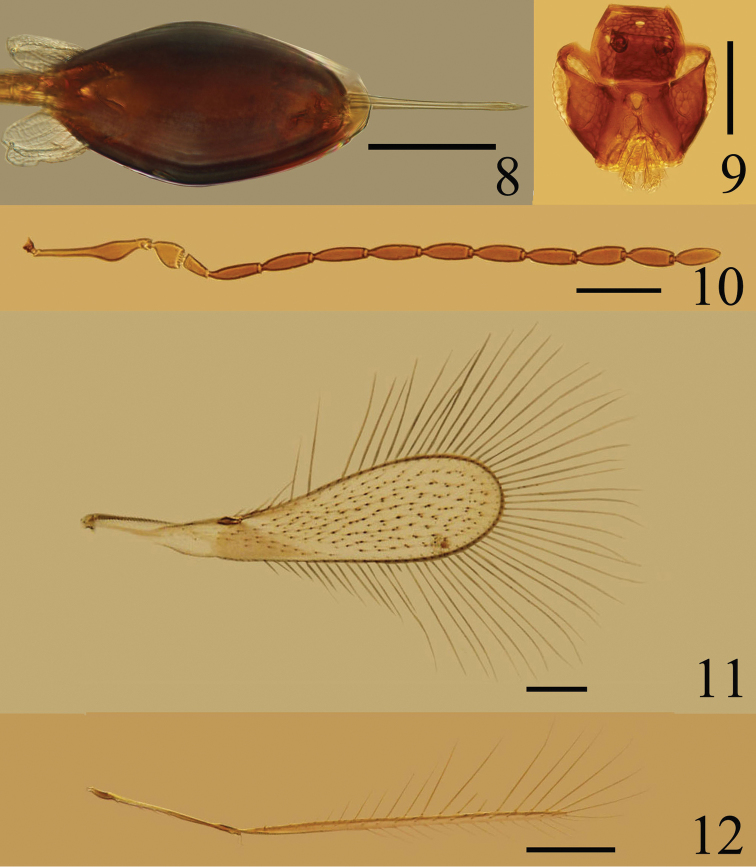
*Ptilomymar dianensis* sp. n., holotype female: **8** gaster. Paratype male: **9** head, dorsal **10** antenna **11** fore wing **12** hind wing. Scale bars=100 μm.

Metasoma. Petiole (Fig. 6) about 2.8× as long as wide. Gaster (Fig. 8) oblong, Gt_1_ (Fig. 7) with 2 large translucent carinae and 1 smaller carinae and a pair of scale-like setae on each side; ovipositor distinctly exserted, about 0.7× as long as mesotibia.

Measurements (length/width, μm): Body length: 500. OD 9.6, OOL 9.6, LOL 33.6, POL 62.4. Antenna: scape 144.0/ 26.4, pedicel 40.8/ 31.2, fl_1_ 26.4, fl_2_ 43.2, fl_3_ 45.6, fl_4_ 38.4, fl_5_ 36.0, fl_6_ 33.6, fl_7_ 33.6, fl_8_ 31.2, clava 136.8/ 55.2. Fore wing 752.4/ 207.9, longest marginal setae 287.1. Propodeum with carinae length 115.2, height 33.6 (measured in lateral view – Fig. 3); gaster with dorsolateral carina length 144, height 67.2 (measured in lateral view – Fig. 7), and ventromedian carina length 120, height 33.6. Ovipositor 201.6.

#### Male.

Similar to female except as follows. Antenna (Fig. 10) with all the flagellar segments longer than wide. Fore wing (Fig. 11) 3.89–4.06× as long as wide. Hind wing (Fig. 12) 0.76–0.78× as long as fore wing, disc with only one row of setae.

Measurements (length/width, μm): Body length 550–580. Antenna: scape 139.2–144.0/ 21.6–26.4, pedicel 43.2/ 28.8–31.2, fl_1_ 33.6, fl_2_ 64.8, fl_3_ 67.2, fl_4_ 38.4, fl_5_ 64.8, fl_6_ 62.4, fl_7_ 62.4, fl_8_ 62.4, fl_9_ 60.0, fl_10_ 60.0, fl_11_ 57.6. Fore wing 643.5–693.0/ 158.4–178.2, hind wing 504.9–524.7.

#### Host.

Unknown.

#### Etymology.

Chinese: dian=Yunnan Province, and refers to the distribution of the species in the Yunnan Province of China.

## Supplementary Material

XML Treatment for Ptilomymar dianensis
